# An Economical, Scaled-Down, Suction-Based Tangential Flow Ultrafiltration System for Biological Process and Product Development Applications

**DOI:** 10.3390/membranes16100321

**Published:** 2026-09-27

**Authors:** Raja Ghosh

**Affiliations:** Department of Chemical Engineering, McMaster University, 1280 Main Street West, Hamilton, ON L8S 4L7, Canada; rghosh@mcmaster.ca; Tel.: +1-905-525-9140 (ext. 27415)

**Keywords:** ultrafiltration, scale-down, tangential flow filtration, biologics, process development, product development

## Abstract

Scaled-down ultrafiltration systems are frequently needed at various stages of protein biologics product and process development. In this paper, an economical and easy-to-use scaled-down tangential flow filtration (TFF) system that is suitable for concentration and buffer exchange of protein solutions, is described and discussed. One or more syringes connected to the permeate side of the membrane module with their plungers pulled out and appropriately clamped were used to generate the pressure drop for filtration by suction. The pumping requirement was therefore significantly less demanding than that in conventional TFF systems. An inexpensive single-purpose pump could easily be used in the proposed TFF system for generating the required tangential flow, as there was no need for generating high pressure on the feed side of the membrane. Also, a feed-side pressure control valve was not required. The hold-up volume of the proposed TFF system was 300 microliters, and feed solutions having volumes as low as 1 mL could easily be processed using it. Its suitability for conducting concentration and buffer exchange experiments was demonstrated by ultrafiltering human immunoglobulin G (HIgG) solutions. An order of magnitude increase in protein concentration (from 10 mg/mL to more than 100 mg/mL) could easily be achieved in 180 min. The highest protein concentration that could be obtained in this study was 152 mg/mL. Buffer exchange of a 50 mg/mL HIgG solution from phosphate buffered saline to low pH acetate buffer and vice-versa could be achieved in 2.5 diafiltration volumes. The specific mechanisms involved in permeate generation in such suction-driven ultrafiltration processes are examined. The role of evaporation under vacuum on the permeate side of the membrane and its potential impact on the process is hypothesized.

## 1. Introduction

Ultrafiltration is a robust and scalable separation technique that is widely used in bioprocessing applications, being extensively used for concentration, desalting, buffer exchange, and diafiltration of biologics [1,2,3]. While the potential for using ultrafiltration for high-resolution purification of biologics has been demonstrated in several studies [4,5,6], its main application in the bio-industry has continued to be in the low-resolution, high-volume bioprocessing segment alluded to above, where it is highly valued for its reliability and reproducibility [7,8]. Tangential flow filtration (or TFF) is the standard method by which ultrafiltration is carried out at an industrial scale [9,10].

One of the recent trends in the area of protein based biotherapeutics such as monoclonal antibodies is the emergence of high-concentration protein formulations (or HCPFs), in which the protein concentration could be greater than 100 mg/mL [11,12,13,14,15]. Some of the direct challenges with HCPFs are those linked to the formulation of such products. The list includes physicochemical factors such as phase separation [16], high viscosity [17], and opalescence [18], as well as stability issues such as propensity to aggregate [19]. HCPFs also generate certain other technological challenges. The manufacturing process for protein biotherapeutics typically involves a TFF-based ultrafiltration/diafiltration (UF/DF) step for adjustment of pH, ionic strength, and the concentration of excipients such as sugars and amino acids [20]. The high protein concentration on the feed side of an ultrafiltration membrane during HCPF production frequently makes it difficult to achieve the target excipient composition and pH in the final product [21,22,23,24]. This is generally attributed to factors such as the Donnan equilibrium effect and volume exclusion effect, both of which are exacerbated at high protein concentrations [21,22,23,24]. This necessitates extensive experimentation during product and process development of HCPFs, particularly for determining appropriate diafiltration buffer composition and processing conditions required for achieving desired targets [21,22,23,24].

Biologics such as monoclonal antibodies are expensive, and limited supplies of these are typically available at the early stages of product and process development. Scaled-down TFF systems are therefore widely used during biologics product and process development [11,25,26]. Most commercial scaled-down TFF systems are simply miniaturized versions of large-scale systems [11,25,26]. While this similitude is useful from a process replication point of view, it creates certain challenges and limitations. In conventional TFF systems, the transmembrane pressure required to drive the filtration process is usually obtained using the combination of an appropriate high-pressure generating pump and a pressure regulating valve on the feed side of the system. This pump also generates the tangential flow within the membrane module, this being necessary to minimize concentration polarization and membrane fouling. Such a requirement for a dual-purpose pump that can not only deliver the required tangential flow but can also generate the required transmembrane pressure adds significantly to the cost of scaled-down TFF systems. The other components of the scaled-down system, such as tubing, connectors, and the membrane module, should also be able to handle such high pressure. Pressure monitoring sensors and shutdown features are typically required in such systems for preventing over-pressurization and for ensuring safety. These additional requirements also contribute significantly to the high cost of currently used scaled-down TFF systems.

Several modified scaled-down membrane filtration systems that mimic or simulate TFF operation are also described in the literature [27,28,29,30,31]. Such systems, in addition to being suitable for routine product and process development applications, are also suitable for process intensification and troubleshooting. An ultra-scale down (USD) approach based on a rotating disc filtration device has been proposed for conducting small-scale membrane filtration [27]. The USD approach has also been used to investigate TFF-based monoclonal antibody processing [28], and for predicting the effects of operating conditions on permeate flux and monoclonal antibody aggregation [29]. A scaled-down cross-flow filtration device consisting of ringed flow channels that is suitable for efficient small-volume microfiltration and ultrafiltration has been described and discussed [30]. More recently, a microfluidic tangential flow filtration device suitable for high throughput process development has been discussed [31]. In all of these systems, the transmembrane pressure was still generated in the conventional way, i.e., by pressurising the feed side of the membrane.

TFF systems, where pumps attached to the filtrate side of the membrane module are used to draw the permeate by suction, have been reported in several studies [32,33,34,35,36,37,38]. One of the main motivations for using suction-driven TFF is to make the transmembrane pressure independent of the tangential flow [32,33,34,35,36,37,38]. Using suction eliminates the need for pressure control valves on the feed side of the TFF system, as the transmembrane pressure could be generated by the permeate pump alone. Also, cascade or multiple-stage ultrafiltration processes are much more efficiently carried out at constant flux conditions using appropriate permeate pumps [33,34,35,36,37]. By using suction-driven ultrafiltration, the protein solution being processed on the feed side of the TFF system remains at a relatively low and consistently more uniform pressure. Also, the absence of a pressure control valve on the feed side could potentially be beneficial from a product stability perspective. The high shear rate and cavitation in pinch points like that within a pressure regulation valve are known to contribute significantly towards protein aggregation and other forms of degradation during TFF [29,39,40]. However, using a permeate pump in an ultrafiltration process comes with one major limitation, i.e., the transmembrane pressure is limited to less than 100 kPa (1 bar).

In this paper, a simple and economical, suction-based, scaled-down TFF system, suitable for biological product and process development applications, is described. The schematic of the system, which consists of a membrane module that was custom-designed for very low hold-up volume [41], is shown in Figure 1. A peristaltic pump was used for generating the tangential flow, and one or more syringes were used for generating the transmembrane pressure required for filtration. These syringes were directly connected to the permeate side of the membrane module as shown in Figure 1, and suction was created by pulling out the plungers to the appropriate extent required and holding them in these positions using plunger clamps (also as shown in Figure 1). The transmembrane pressure was therefore made independent of the tangential flow. Also, valves, pressure sensors, and safety shutdown features were not required. The proposed scaled-down TFF system was used to carry out concentration and buffer exchange of human immunoglobulin G (HIgG) solutions to demonstrate its capabilities. Attributes such as concentration enrichment factor, separation time, permeate flux, recovery, and diafiltration volumes needed for buffer exchange were examined. The results obtained are discussed.

## 2. Materials and Methods

Polyethersulfone (PES) ultrafiltration membrane (Omega, 30 kDa MWCO, part no: OT030SHEET) was purchased from Pall Canada, Mississauga, ON, Canada. Human immunoglobulin G (Human gamma globulin powder, #1860-0047) was sourced from SeraCare, Milford, MA, USA. Sodium phosphate dibasic (S0876), potassium chloride (P9541), and potassium phosphate monobasic (PX1565-1) were purchased from Sigma-Aldrich, St. Louis, MO, USA. Sodium chloride (447300050) was purchased from Thermo-Fisher Scientific Chemicals, Waltham, MA, USA. All buffers and protein solutions were prepared using purified water obtained from a Diamond™ NANOpure (Barnstead, Dubuque, IA, USA) water purification unit. Protein concentration in samples collected during the different experiments was measured at 280 nm wavelength using a NanoDrop 2000c spectrophotometer (Fisher Scientific, Ottawa, ON, Canada). An absorbance versus protein concentration calibration plot was used for measuring protein concentration in samples collected during the TFF experiments. The collected samples were appropriately diluted with process buffer prior to absorbance measurement to bring the absorbance readings to within the linear range of the calibration plots, and this was factored in for determining the actual protein concentration in a given sample.

The ultrafiltration membrane module used for conducting the scaled-down tangential flow filtration experiments in this study (see Figure 2) was fabricated in-house using acrylic. Its design was based on the z^2^LFMC module discussed in a recent paper [41]. This module will be referred to as the z^2^TFF module in this paper. Briefly, it consisted of two acrylic plates with engraved complementary primary and secondary channels (see Figure 2A,B) for enhancing flow distribution on the feed side of the membrane and for enhancing flow collection on the permeate side of the membrane [41]. In addition to enabling flow uniformity, this design also ensured that the dead volume within the membrane module was low [41]. The effective membrane area within the module was 3.35 cm^2^ (1.83 cm × 1.83 cm). The primary channels had a square cross-section (0.5 mm × 0.5 mm), while the secondary channels had a semi-circular cross-section (0.25 mm diameter). The number of secondary channels on each plate was 36. The assembled module with the ultrafiltration membrane in place is shown in Figure 2C. Figure 1 shows the general manner in which the z^2^TFF module was incorporated within the TFF system. The feed solution was introduced into the module using the incoming primary channel on the feed side, from where it was distributed over the surface of the ultrafiltration membrane using the 36 secondary channels. The retentate was collected using the outgoing primary channel on the feed side and directed back to the feed tank. The permeate was collected by suction, using a pair of clamped syringes as shown in Figure 2D.

Figure 3 shows the specific experimental configuration used for conducting the protein concentration experiments with the proposed scaled-down TFF system. A 15 mL Falcon tube, held on a light stand placed on the pan of an electronic balance, served as the feed tank. The screw-cap of the tube was drilled to allow access to light and flexible, small diameter tubing that were used for drawing the feed solution and for returning the retentate solution. The balance was used to track the change in the amount of feed solution within the feed tank during the concentration process. The tubing was selected and arranged such that its weight or rigidity had minimal impact on the reading obtained with the electronic balance. A Reglo Ismatec peristaltic pump (IDEX Corporation, Northbrook, IL, USA) was used for pumping the feed solution into the z^2^TTF module, and thereby generating the tangential flow. There was no pressure control valve in the proposed TFF system, and the retentate was allowed to return to the feed tank without any restriction. The TFF experiments for protein concentration (using 10, 20, and 58 mg/mL HIgG feed solutions) were carried out in triplicate. Figure 4 shows the specific configuration used for conducting diafiltration experiments with the proposed scaled-down TFF system. The diafiltration buffer was held in a separate 15 mL Falcon tube and was fed to the feed tank in a controlled manner using a second peristaltic pump. During the diafiltration experiment, the amount of feed solution within the tangential flow circuit was maintained constant by tracking the reading on the electronic balance and adjusting the flow rate of the second peristaltic pump accordingly. In both concentration and diafiltration experiments, the permeate was drawn by suction using syringes attached directly to the permeate side of the z^2^TFF module (as shown in Figure 3 and Figure 4).

## 3. Results and Discussion

The total hold-up volume on the feed side of the scaled-down TFF system, including the dead volume on the feed side of the module and the total internal volume of tubing, was first experimentally measured by filling up the feed side flow path with buffer and then draining it. This was found to be about 300 μL, out of which about 50 μL was within the module, and the remaining 250 μL could be accounted for in terms of the inner volume of tubing and connector. Such a low hold-up volume enabled the processing of very small feed volumes using this system. The low dead-volume within the custom designed membrane module [41] was primarily due to the use of very shallow semi-circular secondary channels of 0.25 mm diameter.

A protein concentration experiment was first carried out to test whether the proposed scaled-down TFF system was indeed suitable for carrying out protein ultrafiltration. The feed consisted of a 10 mg/mL human immunoglobulin G (HIgG) solution prepared in phosphate-buffered saline (PBS, pH 7.4). The initial volume of feed solution in the Falcon tube used as the feed tank was 10 mL. The tangential flow rate used in this experiment was 4.3 mL/min, while the suction for filtration was generated using two 10 mL syringes, with their plungers clamped in their fully extended position. Figure 5 shows the progression of the HIgG concentration experiment in terms of instantaneous volume of feed solution as a function of time. In this experiment, which was carried out for 180 min, the volume of HIgG solution decreased from 10 mL to about 980 ± 30 μL. The instantaneous volume of solution in the feed tank was calculated based on the mass reading obtained from the electronic balance (0.001 g resolution). The average HIgG concentration in the final solution was found to be 100.5 mg/mL. The average concentration enrichment factor was therefore 10.1, while the average percentage recovery was 98.5%.

Figure 6 shows the volumetric permeate flux (*J_v_*) obtained as a function of time during the HIgG concentration experiment described in the previous paragraph. The *J_v_* data were calculated from the data shown in Figure 5 using the following equation:

*J_v_* = −(1/*A*) (d*V*/d*t*) (1)

where *V* is the volume of liquid in the feed tank, *t* is the time, and *A* is the surface area of the membrane. Polynomial curve fitting (second order) was done to the volume versus time experimental data, and the derivative obtained from it was used to calculate *J_v_*. The permeate flux data calculated using Equation (1) was found to be in good agreement with that obtained based on actual feed volume difference as a function of time in the individual experiments. The decrease in the magnitude of *J_v_* with time can be partly explained by the change in the concentration of HIgG in the feed solution during the TFF-based concentration process. The progressive increase in the HIgG concentration in the feed would result in a progressive increase in the osmotic back pressure (Δ*π*) across the ultrafiltration membrane. Also, with the syringe-based mechanism in the proposed TFF system, the vacuum on the permeate side is expected to decrease somewhat with time. In a trial experiment carried out with a vacuum sensor attached through a T-connector placed between one of the 10 mL syringes and the membrane module, the pressure on the permeate side at the start of the process was found to be 88 kPa below atmospheric pressure. By the end of the 180 min TFF process, the pressure was found to be 79 kPa below atmospheric. This decrease in the extent of vacuum could be attributed to a decrease in available volume on the permeate side due to permeate collection, the evaporation of permeating liquid, and the degassing of the permeate (i.e., removal of dissolved air under vacuum). In the actual TFF experiment (i.e., carried out without the vacuum sensor), the extent of vacuum could be expected to be slightly greater due to the smaller extra-syringe air volume on the permeate side. The pressure on the feed side of the membrane module was unlikely to be affected to a significant effect by the increase in feed viscosity in the HIgG concentration range examined in this experiment. In a trial experiment (i.e., with a pressure sensor connected on the feed line), the feed side pressure was found to be in the 5–10 kPa range. Therefore, the effective transmembrane pressure (Δ*P*) decreased to some extent with time during the concentration experiment. The effect of changes in Δ*π* and Δ*P* on *J_v_* can be explained based on the osmotic pressure model [42]:

*J_v_* = (Δ*P* − Δ*π*)/*R_m_* (2)

where *R_m_* is the membrane resistance. In this specific form of the osmotic pressure model, which is analogous to Ohm’s law used for electric circuits, the permeate viscosity effect is lumped together within the resistance term. The osmotic pressure is generated due to solute concentration difference across the ultrafiltration membrane.

A protein concentration experiment with a higher feed HIgG concentration (i.e., 20 mg/mL, also prepared in PBS, pH 7.4) was carried out. The initial volume of feed solution in the Falcon tube used as the feed tank was 5 mL. As in the previous experiment, the tangential flow rate used in this experiment was 4.3 mL/min, and the suction for filtration was generated using two clamped 10 mL syringes. Figure 7 shows the instantaneous volume of feed solution as a function of time in this concentration experiment, which was carried out for 80 min. The volume of HIgG solution decreased from 5 mL to 905 ± 15 μL. The average measured HIgG concentration in the final solution was 109.1 mg/mL. Therefore, the average concentration enrichment factor was 5.5, while the average percentage recovery was 98.7%. The change in *J_v_* with time during this experiment is shown in Figure 8.

Further increase in target protein concentration was attempted, starting with a feed HIgG solution having a concentration of 58 mg/mL (prepared in PBS, pH 7.4). The initial feed volume was 5.5 mL, while the tangential flow rate used in this experiment was the same as that used in the previous experiments, i.e., 4.3 mL/min. Due to the greater duration of this experiment, suction for filtration was generated using two clamped 20 mL syringes. Figure 9 shows the change in feed volume with time, while Figure 10 shows the change in flux during this experiment, which lasted 210 min. During this time, the feed volume decreased from 5.5 mL to 1.6 ± 0.1 mL. The average concentration of the HIgG solution thus obtained was 152.3 mg/mL, while the average recovery was 76.4%. This reduction in recovery relative to the previous concentration experiments could be attributed to the significant increase in the viscosity of the feed solution. In all the protein concentration experiments, the final enriched solution was recovered simply by taking out the feed tubing from the feed tank while keeping the tangential flow pump running till all liquid was drained out of the tangential flow circuit into the feed tank. Presumably, a more customized procedure for draining the device would result in greater recovery of viscous protein solution. Several strategies for achieving this objective are being studied. The potential role of factors such as protein adsorption and membrane fouling are also being examined.

The buffer exchange experiments were carried out using 50 mg/mL HIgG feed solutions. The HIgG powder was dissolved in the two buffers used in this study, i.e., phosphate-buffered saline (pH 7.4) and 20 mM sodium acetate buffer (pH 5.1). The stable pH obtained by preparing a 50 mg/mL HIgG solution in phosphate buffered saline was 7.35, while the corresponding pH of the 50 mg/mL HIgG solution prepared in acetate buffer was 5.24. The pH shifts observed with the protein solutions relative to their respective buffers could be explained in terms of the self-buffering capacity of HIgG [43], as well as based on the likely presence of residual ionic species in the HIgG powder. Small amounts of salts and other low molecular weight species are typically present in dried IgG products [44].

The first buffer exchange experiment was carried out using the 50 mg/mL HIgG solution prepared using 20 mM acetate buffer (pH 5.1), i.e., having a shifted starting pH of 5.24. The exchange buffer was phosphate-buffered saline (pH 7.4). The volume of feed solution used was 1 mL. The combined hold-up volume of the membrane module and tubing was 300 microliters. Therefore, at any given time during the buffer exchange process, the remaining 700 microliters of solution was in the feed tank. The tangential flow rate used in this experiment was 4.3 mL/min, while the suction for filtration was generated using two clamped 20 mL syringes. The total volume of liquid on the feed side of the membrane module was kept constant by replenishing the liquid lost in the filtrate by pumping in the exchange buffer using a second peristaltic pump (see Figure 4), set at a low flow rate. The mass reading obtained from the balance on which the feed tank was placed (see Figure 4) was used as the basis for keeping the quantity of liquid on the feed side constant. This was quite easy to achieve manually, with only minor adjustments in the flow rate of the second peristaltic pump being required from time to time. The volume of replacement buffer consumed was tracked by observing the volume of liquid remaining in the graduated Falcon tube that was used as the replacement buffer tank (see Figure 4). The buffer exchange process was terminated after 2.5 diafiltration volumes, and the volume, the pH, and the HIgG concentration of the liquid on the feed side of the membrane module were measured. The time required for this buffer exchange process was 68 min. The total volume of buffer exchanged HIgG solution recovered was found to be about 1020 microlitres, its pH was found to be 7.4, while the HIgG concentration was found to be 48.6 mg/mL. The shifted pH that had been obtained while preparing a 50 mg/mL HIgG solution in phosphate-buffered saline (pH 7.4) using HIgG powder was 7.35. Therefore, the above buffer exchange process had clearly removed the ionic species that had been responsible for this slight pH shift.

The second buffer exchange experiment was carried out using the 50 mg/mL HIgG solution prepared using phosphate-buffered saline (pH 7.4), i.e., having a shifted pH of 7.35. The exchange buffer used in this experiment was 20 mM acetate (pH 5.1). As in the previous buffer exchange experiment, the volume of feed solution used was 1 mL. This experiment was carried out in exactly the same way as the previous buffer exchange experiment, i.e., the constant amount of liquid on the feed side was maintained based on the balance reading, and the volume of exchange buffer consumed was monitored based on the liquid level in the Falcon tube. The tangential flow rate used in this experiment was 4.3 mL/min, while the suction for filtration was generated using two clamped 20 mL syringes. The buffer exchange process was terminated after 2.5 diavolumes (62 min), and the volume, the pH, and the HIgG concentration of the liquid on the feed side of the membrane module were measured. The total volume of the buffer-exchanged HIgG solution was found to be about 1040 microlitres; its pH was found to be 5.15, while the HIgG concentration was found to be 47.8 mg/mL. The shifted pH that had been obtained while preparing a 50 mg/mL HIgG solution in 20 mM acetate buffer (pH 5.1) using HIgG powder was 5.24. Therefore, the above buffer exchange process removed some of the ionic species that had been partly responsible for the pH shift. The 0.05 pH unit drift from the target (i.e., pH 5.1) could be explained in terms of the self-buffering property of HIgG at a pH value significantly lower than its isoelectric point (i.e., ~pH 7.0) [43]. Protein retention by ultrafiltration membranes is known to be affected by the pH of the feed solution [4]. While it would be reasonable to expect total retention of HIgG (MW 150 kDa) with a 30 kDa MWCO membrane, the permeate collected in the above buffer exchange experiments were tested to check if they contained any protein. All permeate samples found to be protein-free. In the current study, buffer exchange was assessed based only on the final pH of the feed solution at the end of the process. Additional buffer exchange attributes such as conductivity will be examined in follow-up studies. More robust assessment of buffer exchange involving tests to identify buffer type (i.e., acetate or phosphate) will be carried out in future studies. Buffers containing excipients such as sugars and amino acids will also be examined in such studies.

During suction-based ultrafiltration, the permeate side of the membrane module exists under vacuum. The pressure within a 20 mL suction syringe, clamped under extended conditions, was found to be about 91 kPa below atmospheric pressure at the start of the filtration process. The pressure on the feed side of the membrane module at a tangential flow rate of 4.3 mL/min was found to be in the range of 5 to 10 kPa (due to peristaltic pumping). Therefore, the pressure drop across the membrane was in the range of 96 to 101 kPa at the start of the process. The vacuum on the permeate side of the membrane could be expected to result in the lowering of the boiling point of water contained in the permeate stream. For instance, at 91 kPa vacuum, the boiling point of water is expected to be about 47 degrees Celsius. Some vacuum-induced evaporation of the water present in the permeate stream could therefore be expected. In addition, some degassing of the permeate, i.e., removal of dissolved gases from the liquid phase to the gas phase, could also be expected. This is clearly evident from Figure 11, which shows a photograph of the permeate side of the z^2^TFF membrane module during the above suction-based ultrafiltration processes. The secondary permeate channels visible in Figure 11 were found to contain alternating zones of liquid and gas. The gas phase, which consisted of a mixture of water vapour and air, was present within convex-ended gas slugs, sandwiched between concave-ended liquid slugs. During the filtration process, these gas and liquid slugs moved rapidly within the secondary permeate channels toward the primary permeate channels located on both sides. There, they coalesced with other gas and liquid slugs and formed bigger slugs. Trains of these enlarged gas and liquid slugs then moved toward the permeate side outlets, and ultimately to the space within the suction syringes, where the gas and liquid phases separated into their respective layers.

Figure 12 depicts an asymmetric membrane housed within the z^2^TFF module and regions adjacent to it during the ultrafiltration process. An asymmetric membrane consists of an ultrathin skin layer with fine pores atop a thicker backing layer with pores of significantly larger diameter. In the experiments discussed above, the skin layer faced the feed solution flowing through the secondary feed channels, while the backing layer faced the secondary permeate channels. The permeate flowed within the membrane pores from the feed side, which was at a slightly higher pressure than atmospheric, to the permeate side, which was under vacuum. As depicted in Figure 12, some evaporation of water could be expected to occur on the permeate side of the membrane due to the lowering of the boiling point under vacuum. Such vacuum-induced evaporation of permeating liquid on the permeate side of the membrane could potentially constitute a secondary mechanism for promoting permeate generation during suction-based ultrafiltration. This hypothesis will be examined in detail in future studies. Also, such vacuum-induced evaporation is likely to result in a localized increase in the concentration of membrane-permeable solutes on the permeate side of the membrane. Whether such localized enrichment of permeable solutes has any impact on the filtration process will be examined in future studies. In conventional pressure-based ultrafiltration, both sides of the membrane are liquid-filled. Therefore, colligative phenomena such as osmotic pressure are able to affect the transport of liquid through the membrane [42], and electrostatic phenomena such as the Donnan equilibrium effect [45] are able to influence the transport of charged species through the membrane. However, in suction-based systems, the development of bubbles and gas slugs on the permeate side of the membrane (see Figure 11 and Figure 12) results in sporadic disruption of liquid continuity across the membrane. Whether such disruption has any impact on the osmotic pressure or on the Donnan equilibrium effect will be systematically investigated in future studies.

The above results clearly demonstrate that the proposed scaled-down suction-based TFF system was suitable for carrying out concentration and buffer exchange of protein biologics. By extension, it is also likely to be suitable for operations such as desalting and removal of small molecular weight impurities from protein solutions. However, such suction-based ultrafiltration is unlikely to be suitable for processes where the product species is present in the permeate stream. The spontaneous solvent evaporation under vacuum is likely to lead to alteration in permeate composition, which would be detrimental to the product quality in applications where the target product is present in the permeate. Also, the formation of bubbles could lead to damage to the product on the permeate side through mechanisms such as denaturation and aggregation. However, in the vast majority of biological applications of ultrafiltration where the product species usually remain on the feed side, such bubble formation on the permeate side would have no negative impact on either product quality or stability. The proposed scaled-down suction-based TFF system is based on the use of pulled-out and plunger-clamped syringes. A suction-driven, commercial TFF system developed based on this operating principle is likely to be significantly cheaper than conventional positive pressure-based TFF systems, which typically cost several tens of thousands of US dollars. The anticipated cost of a syringe-based, suction-driven TFF system put together using an inexpensive feed pump and accessories would be several thousand US dollars at the most. While the proposed system is likely to be economical and convenient to use at a laboratory scale, the finite volume of the syringe would limit the extent and duration of the vacuum generated, which would in turn place limits on the volume of material that could be processed. This limitation could be addressed by using permeate pumps to generate more sustained suction, or indeed by connecting the permeate side to some other sustained vacuum source, such as a vacuum pump. The minimum volume of feed solution that could be processed using the proposed syringe-based, suction-driven TFF system was comparable to typical minimum volumes for miniaturized commercial TFF systems [26]. Future work will explore further miniaturization using the syringe-based approach. At the same time, scale-up of suction-based systems incorporating appropriate permeate pumps will also be looked at. Aspects such as protein adsorption, aggregation, and leachables released from membrane modules will be examined in future studies.

## 4. Conclusions

The suitability of using the proposed scaled-down, suction-based TFF system for concentration and buffer exchange of human immunoglobulin G (HIgG) solutions is demonstrated in this paper. The proposed system is economical and easy to assemble and operate. The suction required for driving filtration was created by using one or more syringes with their plungers clamped in their extended positions. Due to the use of suction-driven filtration, the feed side did not need to be pressurized. Hence, an inexpensive single-purpose peristaltic pump could easily be used to generate the tangential flow. Also, a pressure regulation valve was not required on the feed side of the proposed TFF system. Both these features are likely to make the proposed suction-based TFF system significantly cheaper than conventional positive-pressure-based TFF systems. The elimination of the pressure regulating valve is also likely to result in less protein denaturation and aggregation, which typically take place in pressure pinch-points such as those within pressure controllers. However, the main limitation of using syringes for generating suction is a finite processing capacity. Therefore, this approach would only work for miniaturized TFF systems. The dead volume on the feed side of the TFF system was very low (300 microlitres) and hence very small volumes of feed solution (in the range of 1 mL) could easily be processed using the proposed system. More than ten-fold enhancement in HIgG concentration was obtained when the proposed scaled-down TFF system was operated in the concentration mode, the resultant protein concentration being in excess of 100 mg/mL. A 58 mg/mL HIgG feed solution could be further enriched to 152 mg/mL concentration in 210 min. However, the protein recovery decreased with an increase in solution viscosity, and an appropriate recovery strategy would therefore be required for obtaining very highly concentrated protein solutions. Buffer exchange of HIgG solution could be achieved in 2.5 diafiltration volumes. The permeate side of the membrane was found to contain alternating gas and liquid slugs. The mechanism of gas slug formation by evaporation under vacuum conditions is explained, and its potential impact on the filtration process is hypothesized. The formation of bubbles on the permeate side is not a detrimental issue when the product remains on the feed side of the membrane, such as in the applications studied in this paper. However, suction-based ultrafiltration would not be suitable for applications where the product is present in the permeate stream.

## Figures and Tables

**Figure 1:**
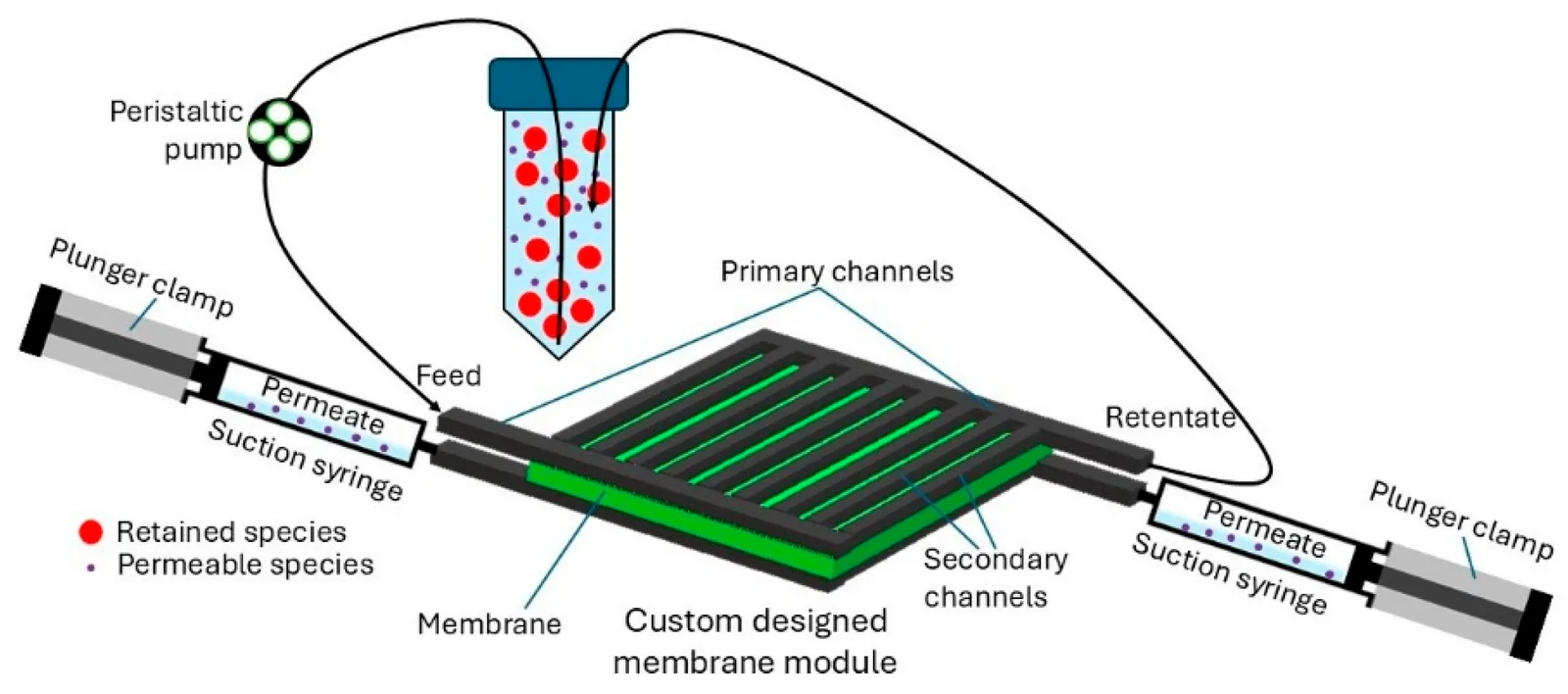
Schematic diagram showing the workings of the proposed scaled-down, suction-based tangential flow filtration system incorporating a custom-designed membrane module.

**Figure 2:**
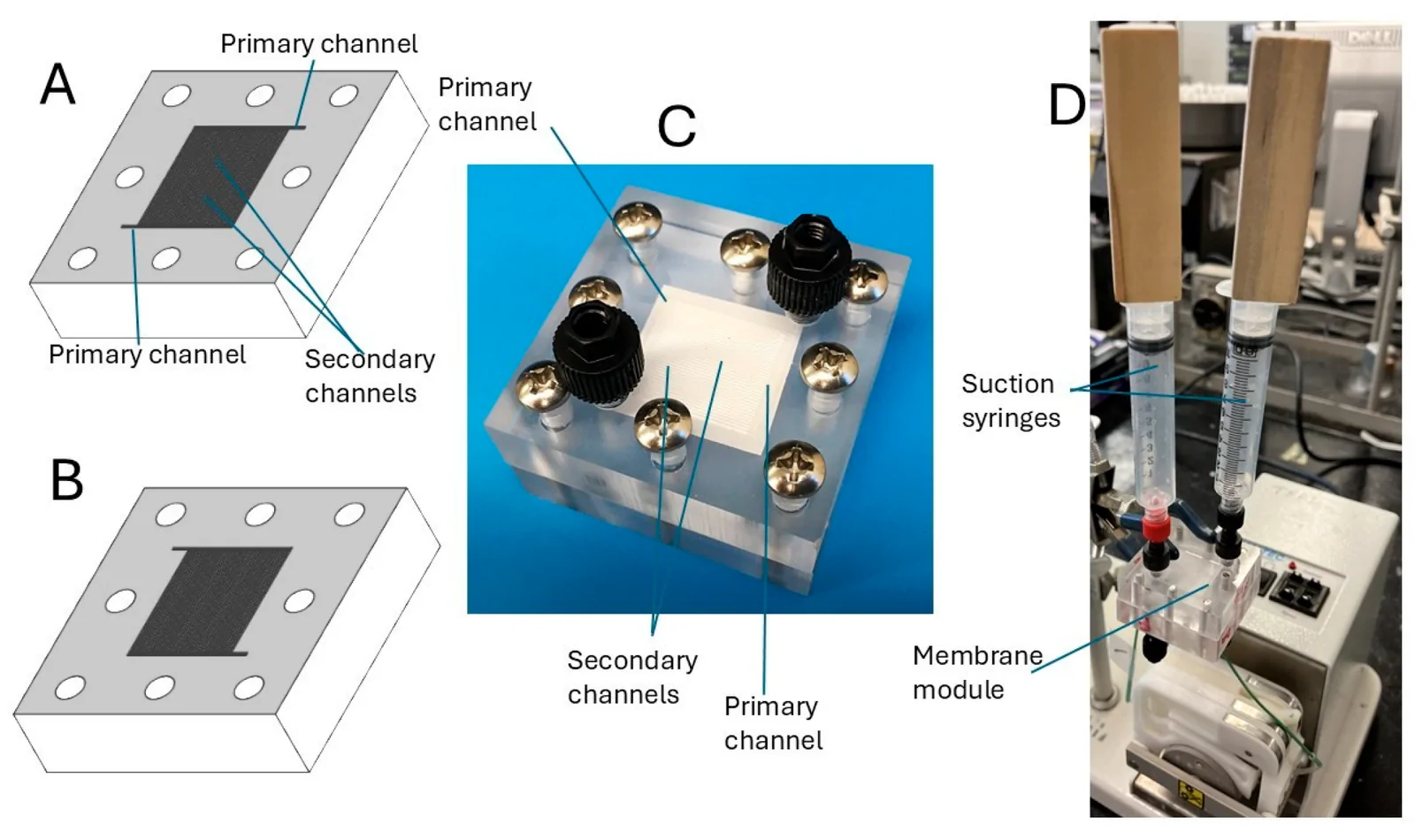
Membrane module used for scaled-down tangential flow filtration experiments ((**A**) feed distribution plate, (**B**) permeate collection plate, (**C**) assembled z^2^TFF module, (**D**) attachment of suction syringes to the z^2^TFF module).

**Figure 3:**
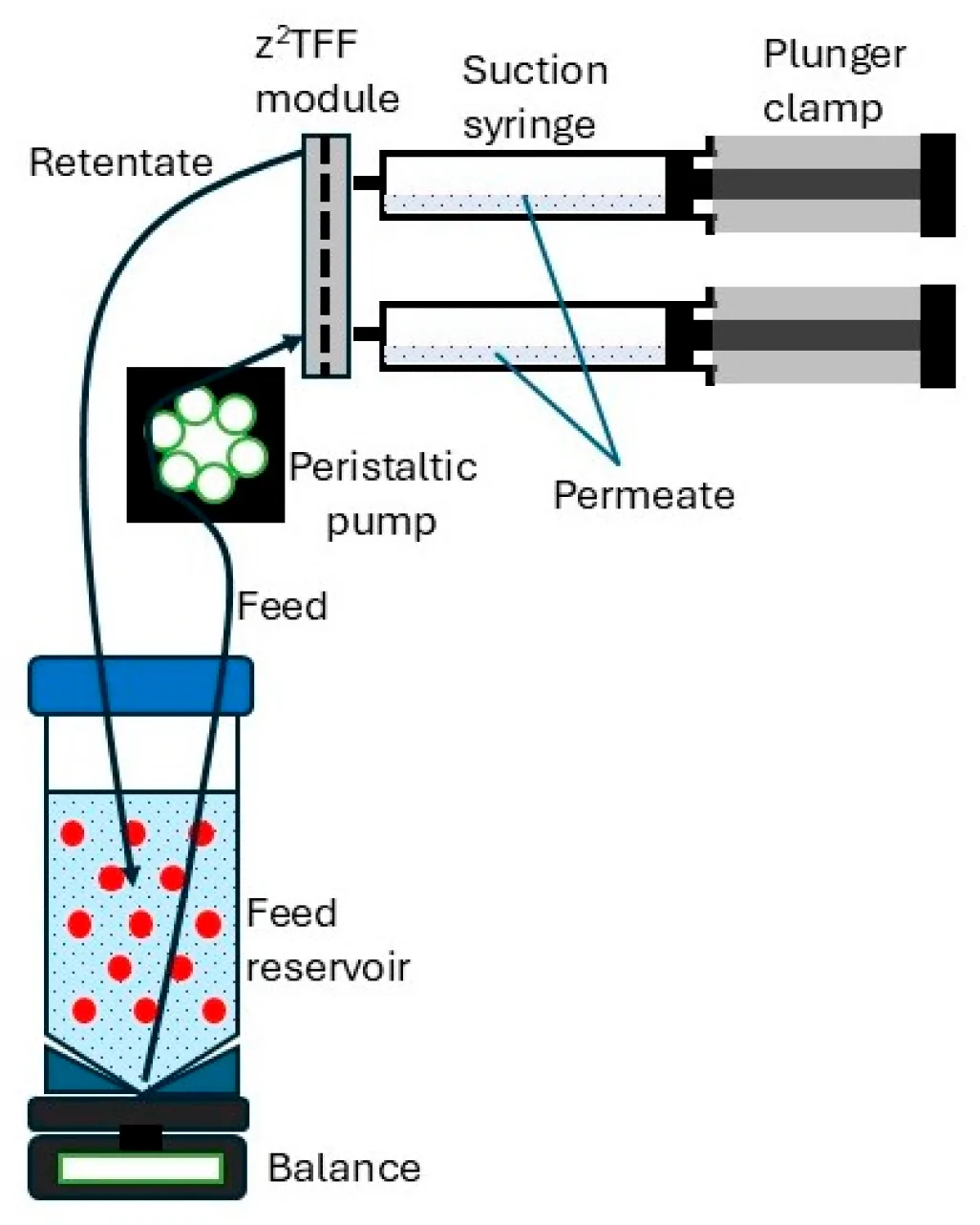
Experimental set-up for conducting concentration experiments using the proposed scaled-down TFF system.

**Figure 4:**
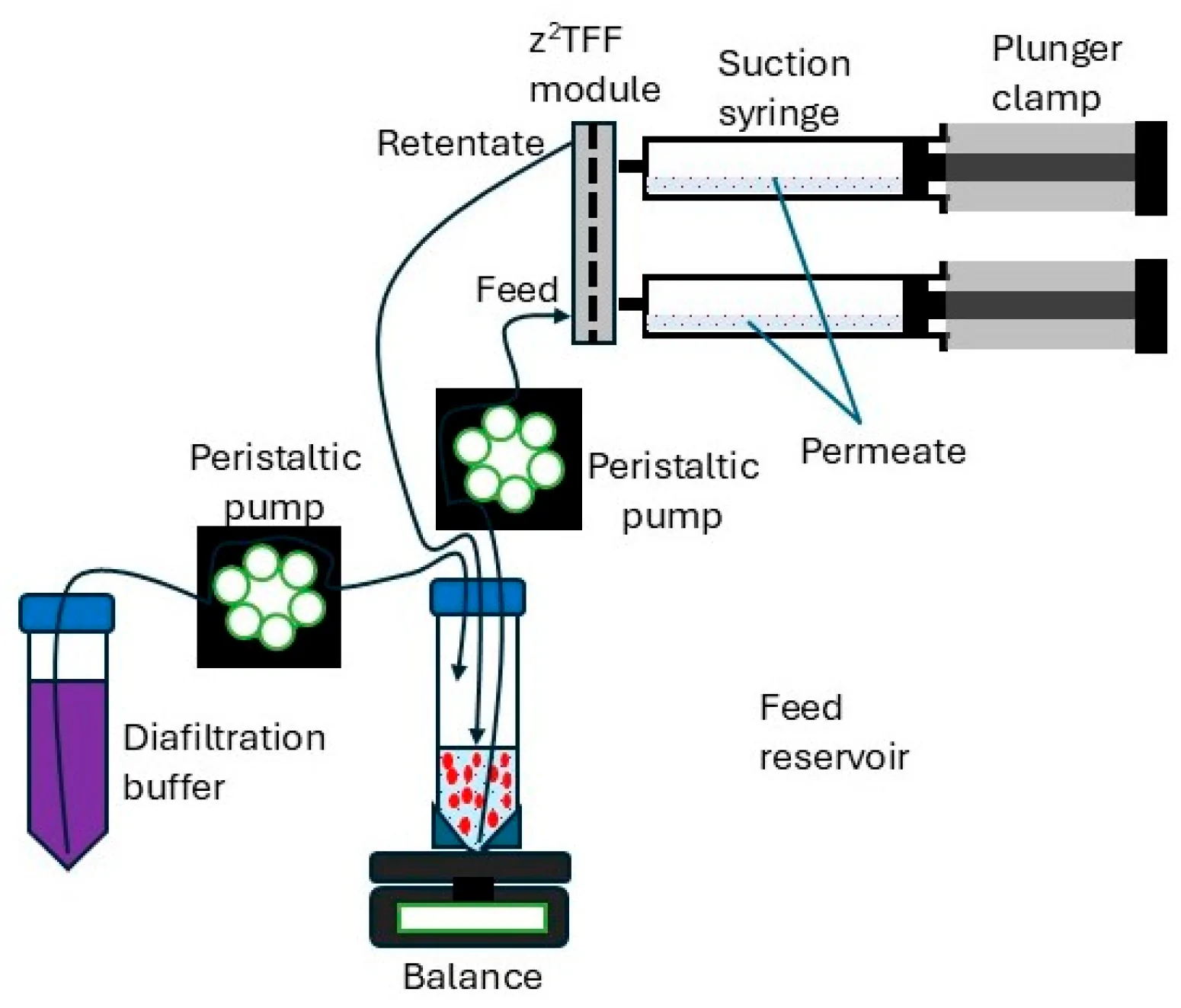
Experimental set-up for conducting diafiltration experiments using the proposed scaled-down TFF system.

**Figure 5:**
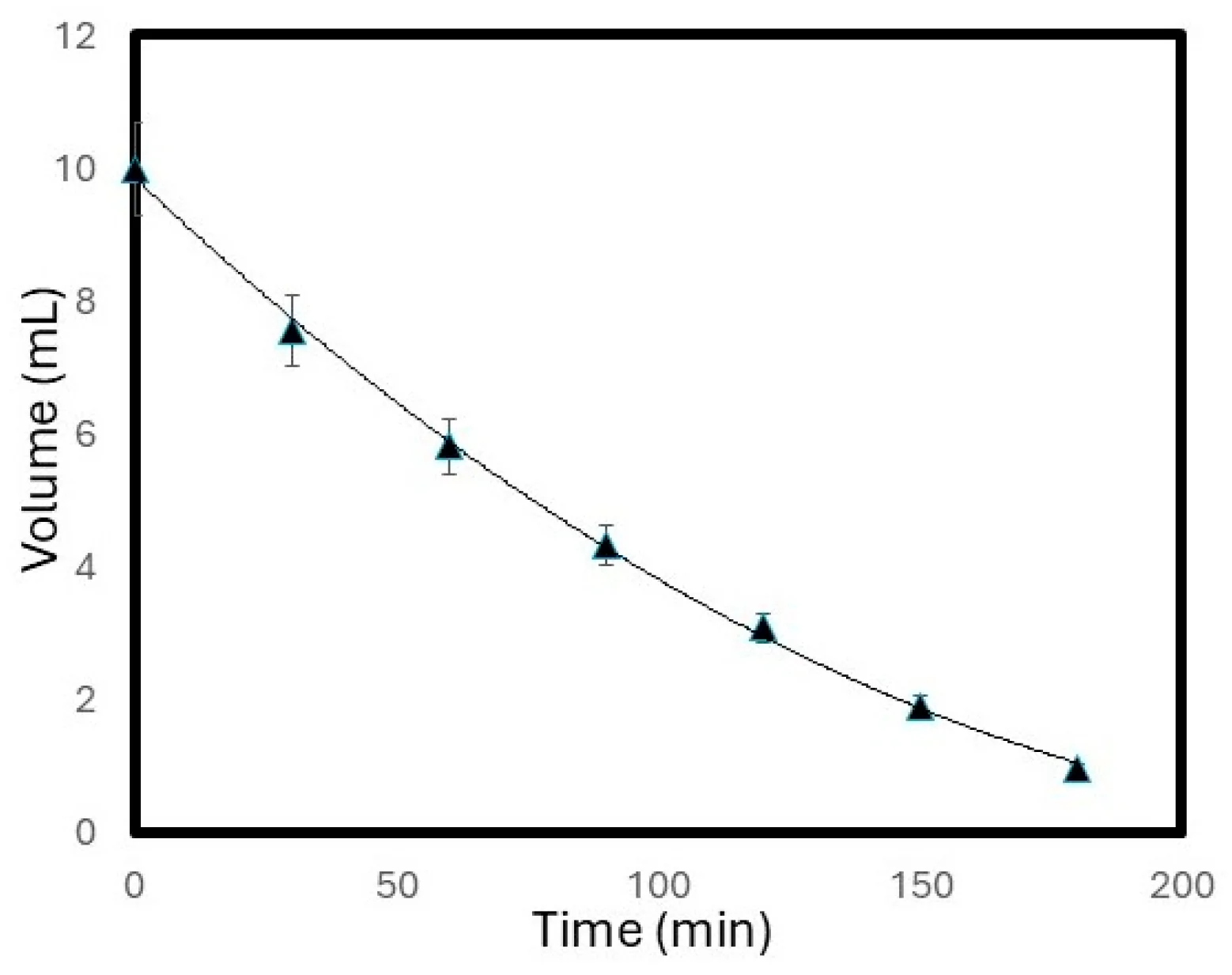
Volume of feed as a function of time during concentration of 10 mg/mL feed HIgG solution.

**Figure 6:**
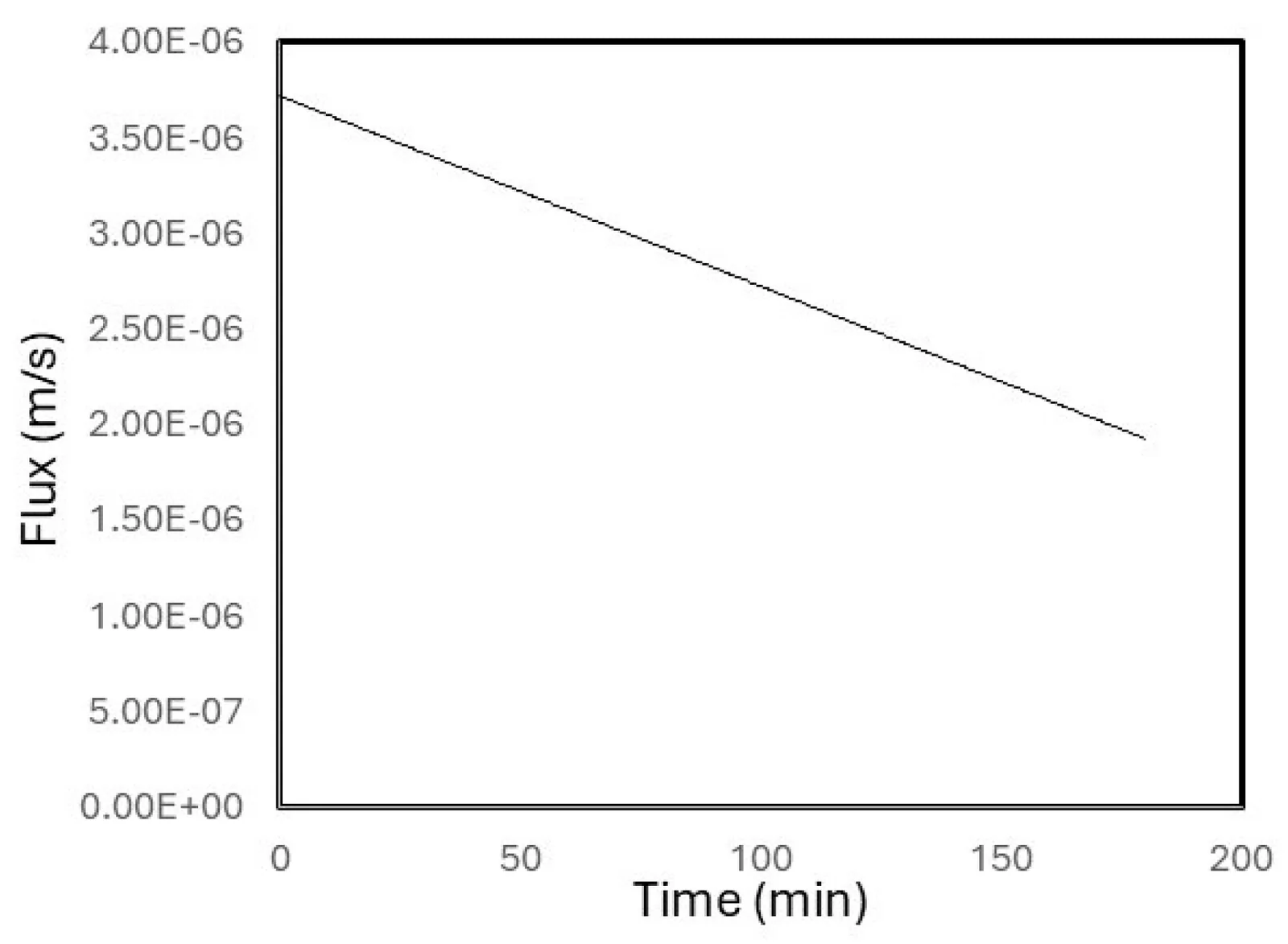
Volumetric permeate flux as a function of time during the concentration of 10 mg/mL HIgG feed solution.

**Figure 7:**
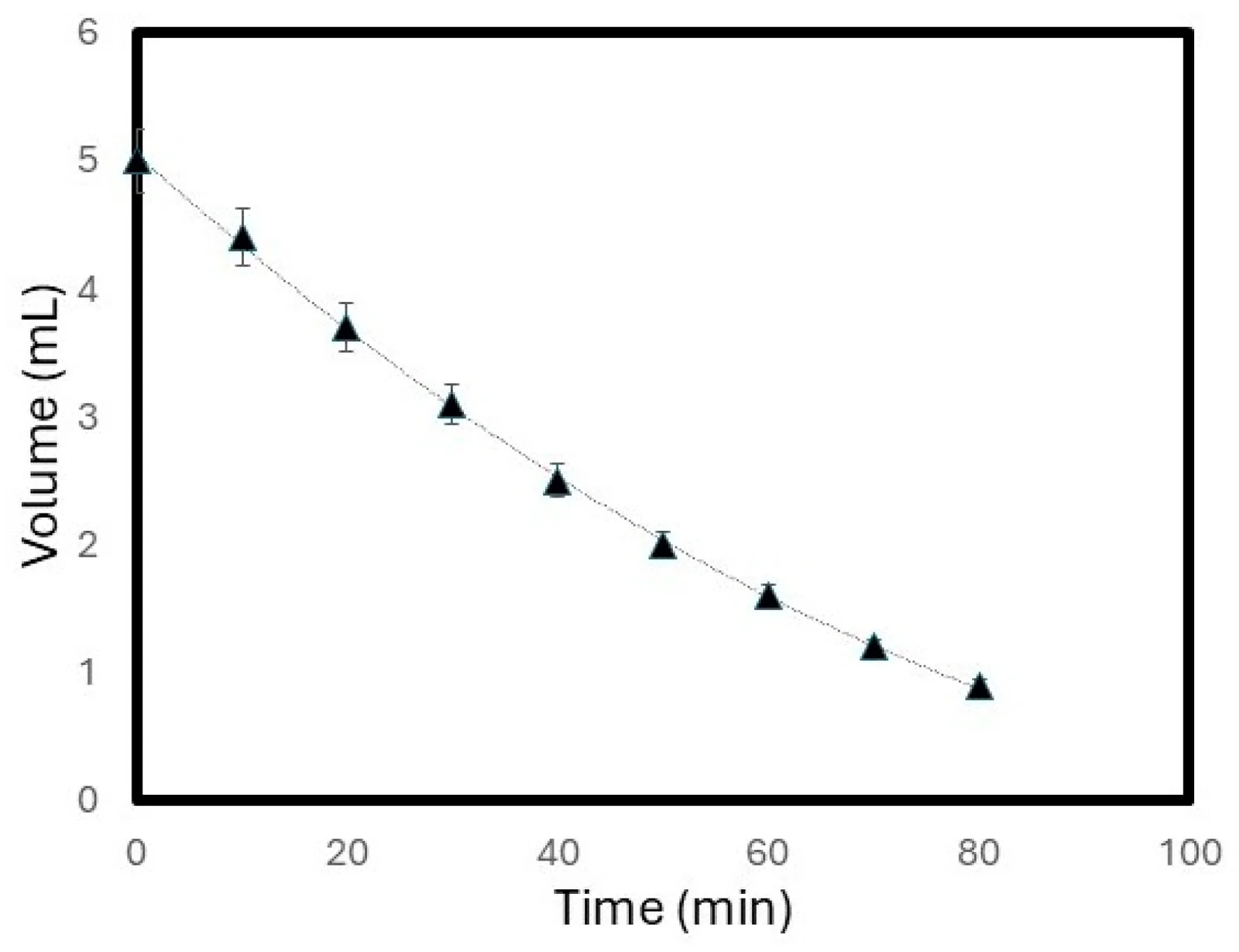
Volume of feed as a function of time during concentration of 20 mg/mL HIgG feed solution.

**Figure 8:**
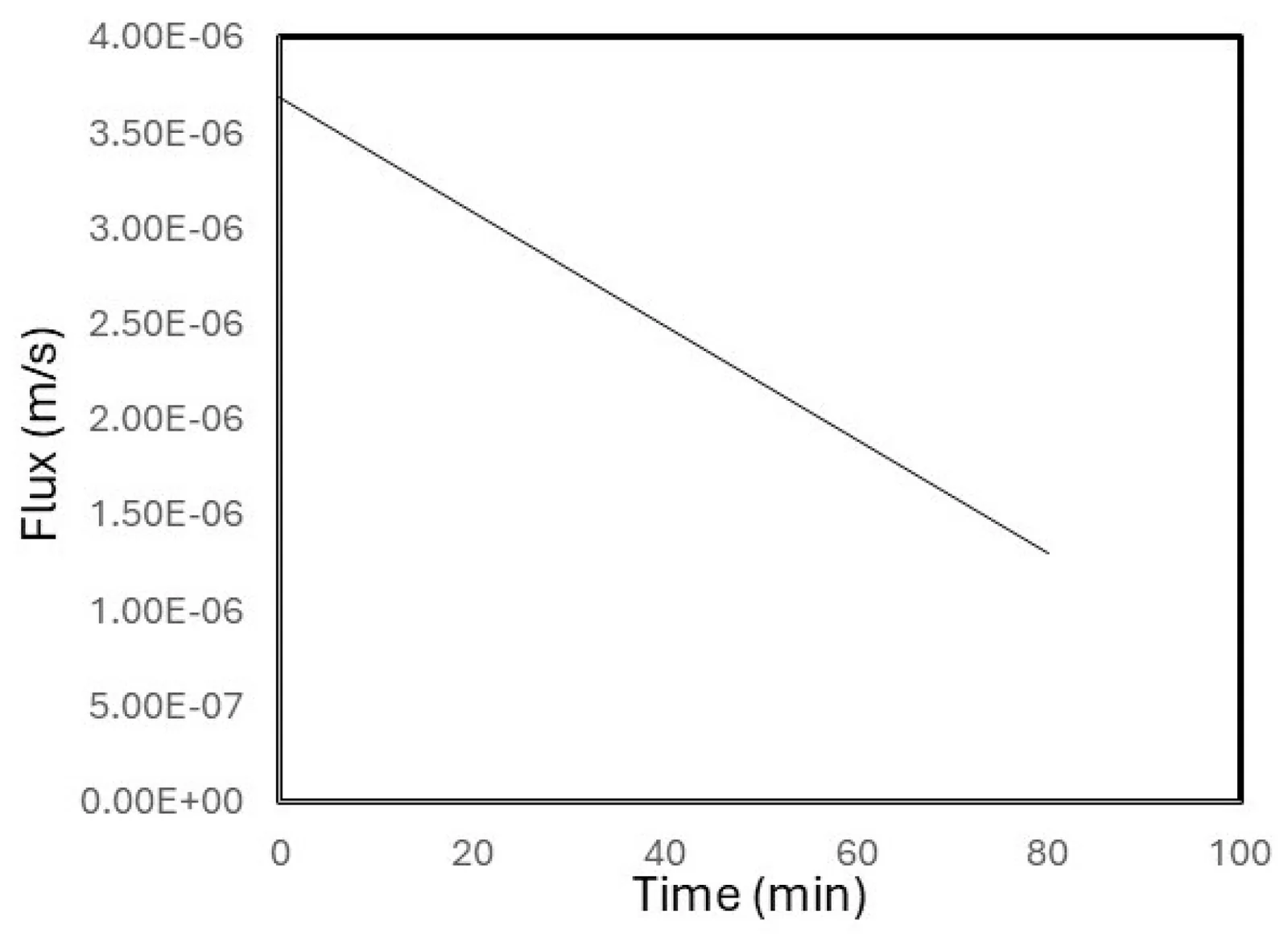
Volumetric permeate flux as a function of time during concentration of 20 mg/mL HIgG feed solution.

**Figure 9:**
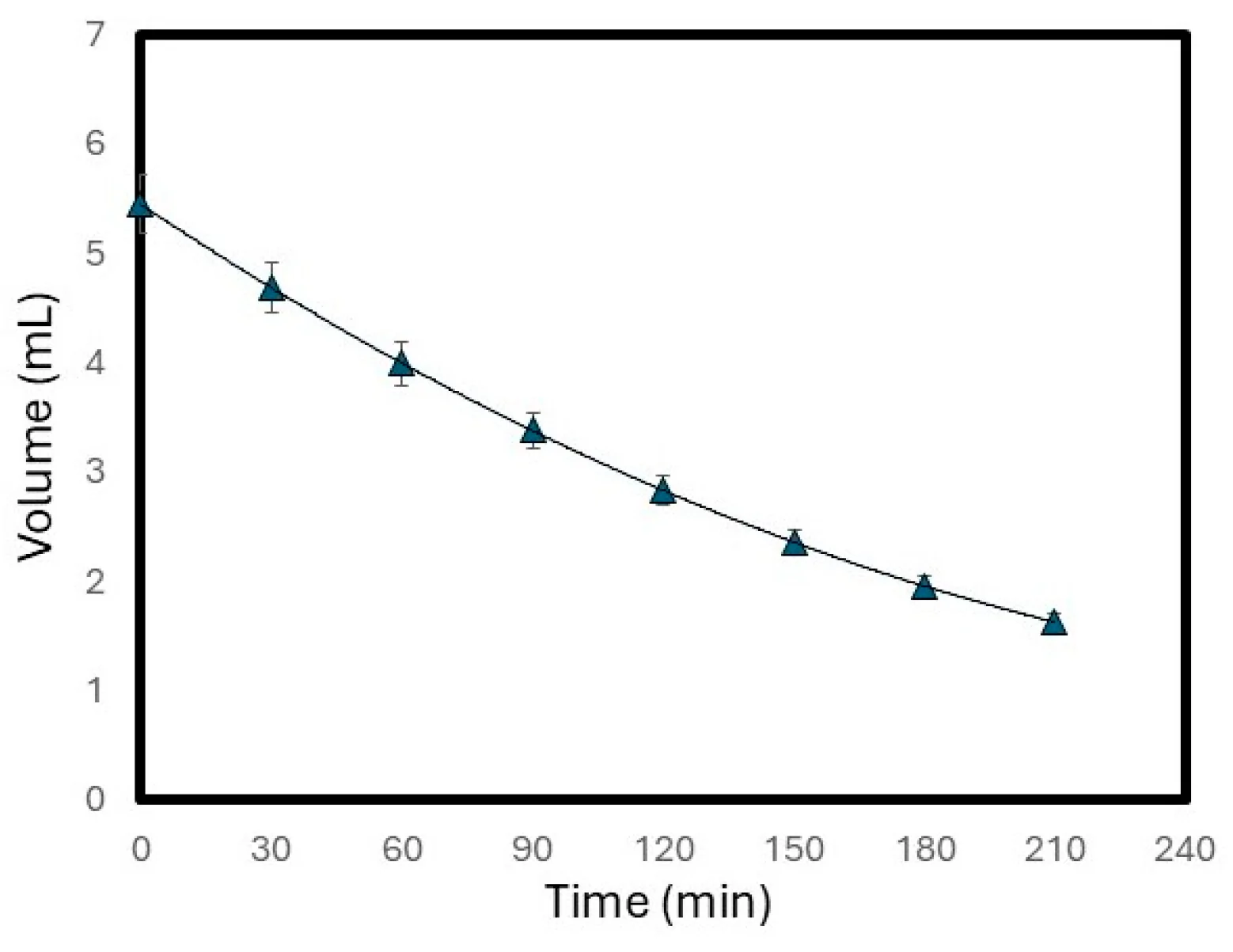
Volume of feed as a function of time during concentration of 58 mg/mL HIgG feed solution.

**Figure 10:**
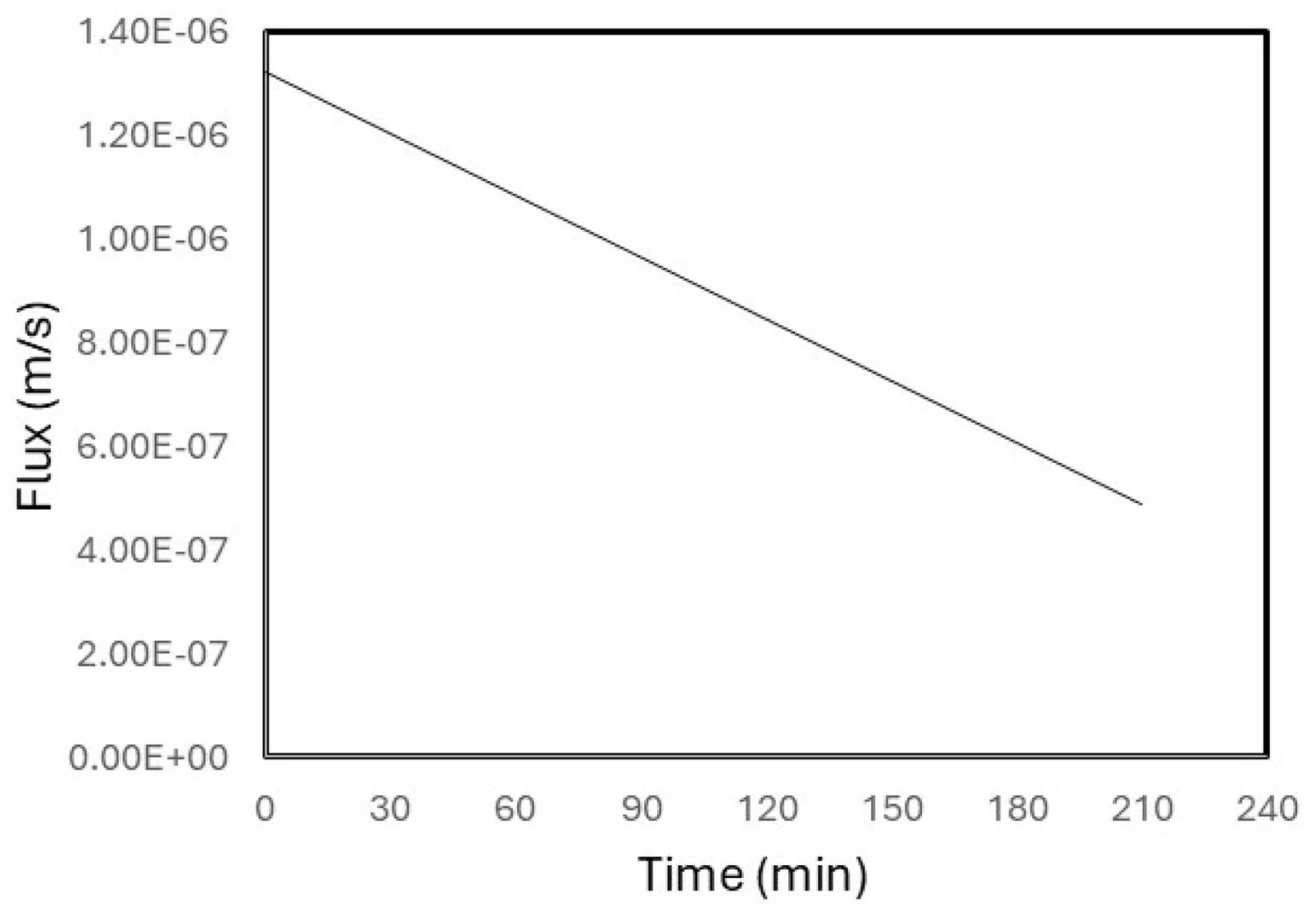
Volumetric permeate flux as a function of time during concentration of 58 mg/mL HIgG feed solution.

**Figure 11:**
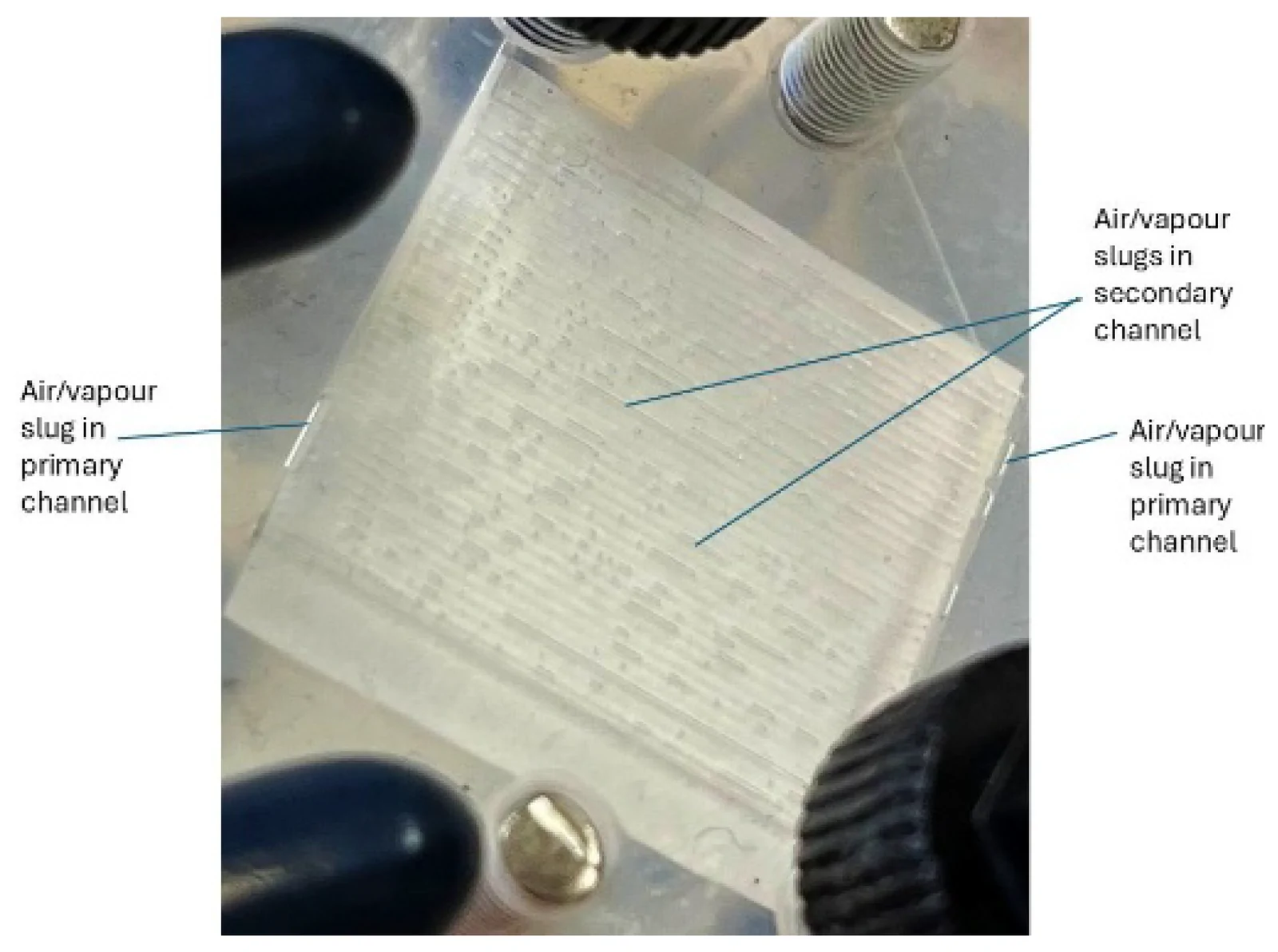
Appearance of gas and liquid slugs in the collecting channels located on the permeate side of the membrane in the z^2^TFF module during suction-based ultrafiltration.

**Figure 12:**
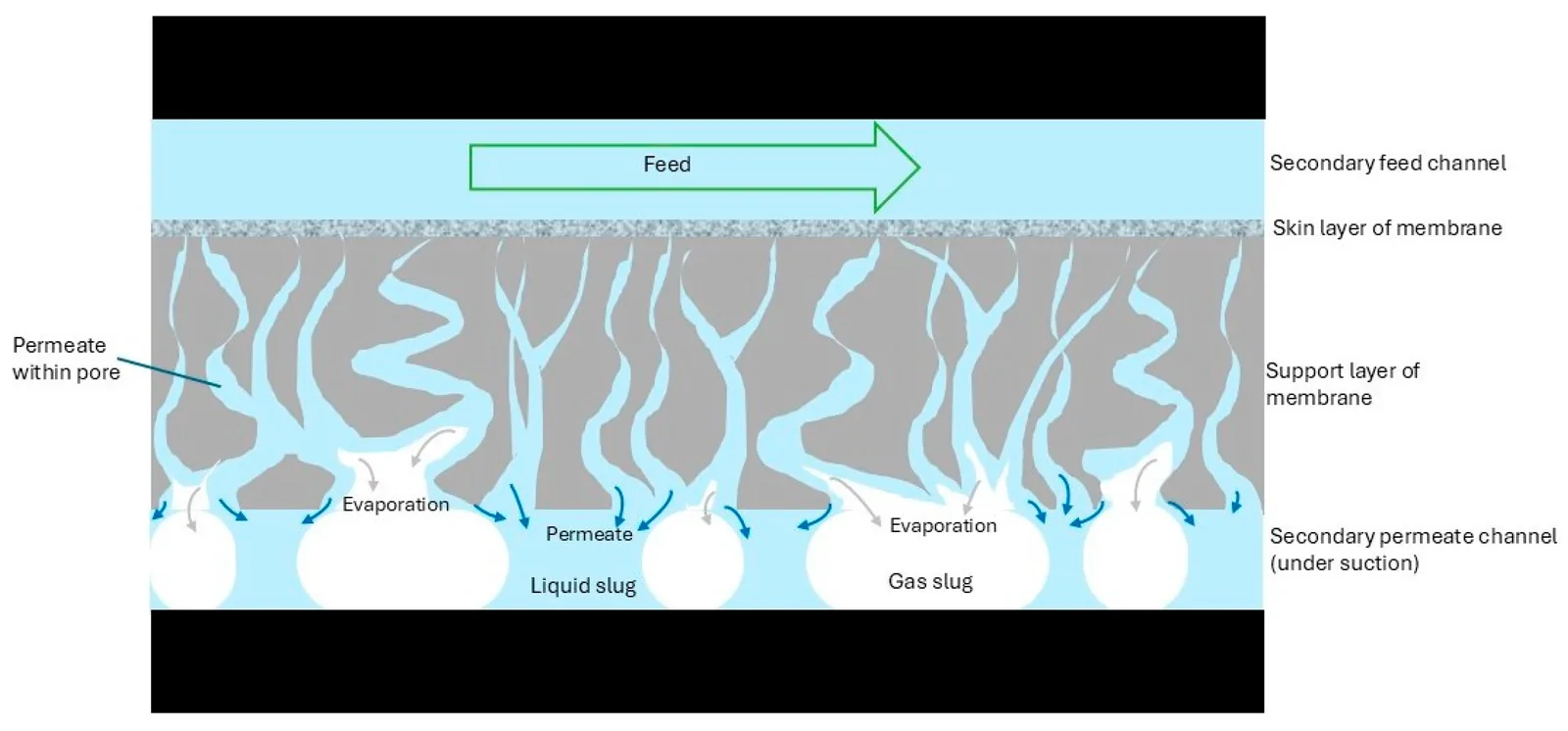
Mechanism of formation of gas and liquid slugs on the permeate side of the z^2^TFF module during suction-based ultrafiltration.

## Data Availability

The original contributions presented in the study are included in the article. Further inquiries can be directed to the corresponding author.
